# Mucinous Differentiation in Colorectal Cancer: A 10-Year Experience Audit at King Faisal Specialist Hospital and Research Centre, Jeddah

**DOI:** 10.7759/cureus.56722

**Published:** 2024-03-22

**Authors:** Khalid A Alshehri, Naif Alsulaimani, Wejdan A Alghamdi, Zuhoor Almansouri, Syed A Zubair, Jamal Zekri, Haitham Saimeh, Sufian Sultan

**Affiliations:** 1 Surgery, King Faisal Specialist Hospital and Research Centre, Jeddah, SAU; 2 General Surgery, King Faisal Specialist Hospital and Research Centre, Jeddah, SAU; 3 Anatomic Pathology, King Faisal Specialist Hospital and Research Centre, Jeddah, SAU; 4 Medical Oncology, King Faisal Specialist Hospital and Research Centre, Jeddah, SAU; 5 Oncology, King Faisal Specialist Hospital and Research Centre, Jeddah, SAU; 6 Medicine, Al-Faisal University, Jeddah, SAU

**Keywords:** gi oncology, oncology, chemo radiotherapy (chemo-rt), colorectal cancer surgery, colorectal surgery, surgery

## Abstract

Given that colorectal cancer is one of the leading causes of mortality, mucinous adenocarcinoma is one of the subtypes and is characterized by the presence of mucin-producing tumor cells with mucin components and is more challenging to manage. In Saudi Arabia, it represents approximately 10-15% of all colorectal carcinoma. The main etiological cause of mucinous adenocarcinoma is yet not well understood. The main goal of our study is to discuss the histopathology and the molecular background of mucinous colorectal adenocarcinoma and also to provide an update on its prognosis and therapeutics from recent published literature. It is a retrospective cohort study that was conducted at King Faisal Specialist Hospital, Jeddah, Saudi Arabia. The study included 68 adult patients diagnosed with mucinous colon cancer, who did surgical resection alone or with or without adjuvant chemotherapy following from January 2011 to December 2020. The mucinous subtypes are found more commonly in the proximal colon. In our study, 26 patients (38.2% of the cases) were right-sided and 35 patients (51.5%) were from the left side, but these included the rectum as well and this reflects the higher incidence of diagnosis of rectal cancer in the region. Most tumors were classified as Grade II in 56 patients (82.4%), consistent with the intermediate differentiation status often associated with the mucinous subtypes. The most common symptom at presentation was abdominal pain in 38 patients (55.9%) followed by per rectal bleeding and abdominal mass. The management in our study was in line with the standard established practice and surgical resection as expected was the primary potentially curative approach. Notably of patients presenting with locally advanced rectal cancer, six patients underwent concomitant chemoradiotherapy followed by surgery and four patients had upfront surgery. The duration of the median follow-up was 32 months. At the time of analysis, 30 patients (44.1%) were alive and remained on regular follow-up, 17 patients (25%) had succumbed to the disease, and 21 patients (30.9%) were lost to follow-up. The median overall survival was not reached, and notably, 49 patients (71.6%) remained alive at the four-year mark. Whilst our study contributes to the current understanding of mucinous adenocarcinomas of the colon, further research in molecular profiling and genomic testing and larger clinical trials with tailored treatments is necessary to refine treatment strategies and improve outcomes.

## Introduction

Colorectal cancer is one of the leading causes of mortality [1]. One of the distinct subtypes, mucinous adenocarcinoma characterized by the presence of mucin-producing tumor cells with a mucin component being at least 50% of the tumor volume has very distinct clinical and pathological features and is more challenging to manage [2].

Worldwide, colorectal cancer is one of the major driving causes of cancer-related death. In Saudi Arabia, colorectal cancer tends to present among young, aged individuals, mainly females and is a major burden on healthcare facilities. The mucinous colorectal cancer histological subtype of adenocarcinoma tends to represent approximately 10-15% of all colorectal carcinoma and is characterized by the predominant histological pattern of extracellular mucin [3].

Mucinous adenocarcinoma is an unfavorable subtype of colon cancer with unique distinct clinical histopathological characteristics due to different microsatellite instability, mucin is a target for molecular therapy with specific genetic mutation. Mucinous tumors are highly associated with a greater proportion of patients having both nodal as well as peritoneal metastases, on the other hand, the liver is the most common site of metastases for patients having non-mucinous histology [4].

Mucinous adenocarcinoma is characterized to have a worse prognosis compared to non-mucinous adenocarcinoma taking into consideration that the prognostic factors are constant [5], since after chemotherapy together with radiotherapy-based treatment the tumor downstaging, as well as the pathological complete response is worse in contrast to the non-mucinous type. This arising resistance may be due to gene mutations.

The main etiological cause of mucinous adenocarcinoma is yet not well understood; however, certain recent studies found that genetic mutations including KRAS, BRAF mutation as well as microsatellite instability altered and influenced by the main diet rich in fat, and low fibers in addition to sedentary lifestyle habits including the absence of sufficient physical exercises, smoking, and alcohol consumption. However, the absence of the risk factors does not mean the absence and exclusion of the disease, always patient presentation in addition to the clinical findings should be taken into consideration [6].

The main aim of our study is to discuss the histopathology and the molecular background of mucinous colorectal adenocarcinoma and also to provide an update on its prognosis and therapeutics from recent published literature.

## Materials and methods

Patients and statistical analysis

This retrospective cohort study was conducted at King Faisal Specialist Hospital, Jeddah, Saudi Arabia in the medical record department and, in oncology and surgical wards. The study data was obtained from the archives of the cytology laboratory and the health information system in our hospital "Powerchart," which were verified by histopathological diagnosis. The study included adult patients (both genders) from January 2011 to December 2020. Only cases with the diagnosis of mucinous carcinoma or adenocarcinoma with mucinous differentiation were included in the review. Low-grade appendiceal mucinous neoplasm (LAMN) and high-grade appendiceal mucinous neoplasms (HAMN) were not included. The slides of the cases with the diagnosis of adenocarcinoma with mucinous differentiation were reviewed to estimate the percentage of mucinous differentiation. The slides of the cases with histological grade (moderate to poorly differentiated) were reviewed to determine the grade according to the percentage of glandular differentiation. The study included patients who were diagnosed with mucinous colon cancer, and who did surgical resection alone or with or without adjuvant chemotherapy from January 2011 to December 2020. Patients with other types of colorectal cancer were excluded. Further exclusion was done by the oncology team depending on the available data and follow-up. The sample size includes 68 patients randomly selected in the study area. Data were analyzed using IBM SPSS Statistics for Windows, version 23.0 (IBM Corp., Armonk, NY). The margin of error was 5%, with a 95% confidence level. This study was approved by the institutional review board and research ethics committee of King Faisal Specialist Hospital and Research Centre, Jeddah, and was given registration number H-002J-009.

Data collection method

The study data were collected using a data collection sheet after reviewing multiple data collection sheets in many studies that study the same topic as our article. The data collection sheet that was used in our study consists of six parts. The first part collected basic information about patients including medical record number, gender, age at diagnosis, presentation, and tumor site. The second part collected tumor staging including tumor differentiation and TNM (tumor, node, metastasis) staging. The third part collected management whether surgical and/or chemotherapy. The fourth part collected molecular markers. The fifth part included the recurrence of the tumor and relapse-free survival in months. The last part includes patient follow-up status and overall survival. Patient names were not collected to maintain confidentiality.

## Results

Sixty-eight patients with adenocarcinoma with mucinous components of colorectal origin were identified and are the subject of this analysis. Males and females constituted 37 (54.4%) and 31 (45.6%) of the cohort and the median age was 59 (21-89) years (Table 1).

**Table 1:** Patients and tumor characteristics. AJCC: American Joint Committee on Cancer.

Patients and tumor characteristics	Number (%)
Gender	
Male	37 (54.4%)
Female	31 (45.6%)
Median age (range)	59 (21-89) years
Primary presenting symptoms	
Abdominal pain	38 (55.9%)
Rectal bleeding (fresh or melena)	29 (42.6%)
Abdominal swelling (mass)	1 (1.47%)
Tumor site within the colorectal tract	
Right side	26 (38.2%)
Transverse colon	5 (7.4%)
Left side (including rectum)	35 (51.47%)
More than one site	2 (2.9%)
Tumor grade	
Grade I	3 (4.4%)
Grade II	56 (82.4%)
Grade III	9 (13.2%)
AJCC tumor stage at diagnosis	
Stage I	5 (7.35%)
Stage II	18 (26.47%)
Stage III	36 (52.94%)
Stage IV	9 (13.23%)
Lymphovascular invasion	
Present	22 (32.4%)
Absent	46 (67.6%)

In general, the management of patients followed standards of treatment of colorectal cancer. Concomitant radio-chemotherapy followed by surgery was the primary treatment for six patients with locally advanced rectal cancer. The other four patients with rectal cancer underwent upfront surgery.

**Figure 1:**
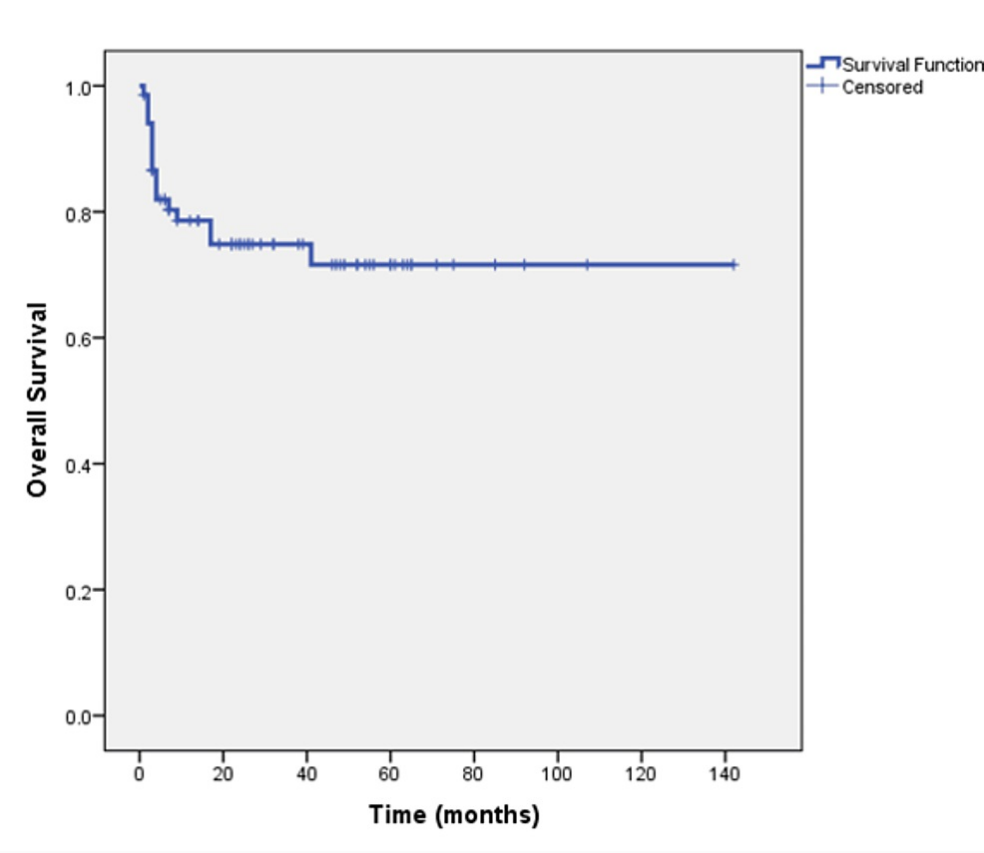
Overall survival of all patients (n=68 patients) with all stages (I/II/III/IV) at the time of presentation.

**Figure 2:**
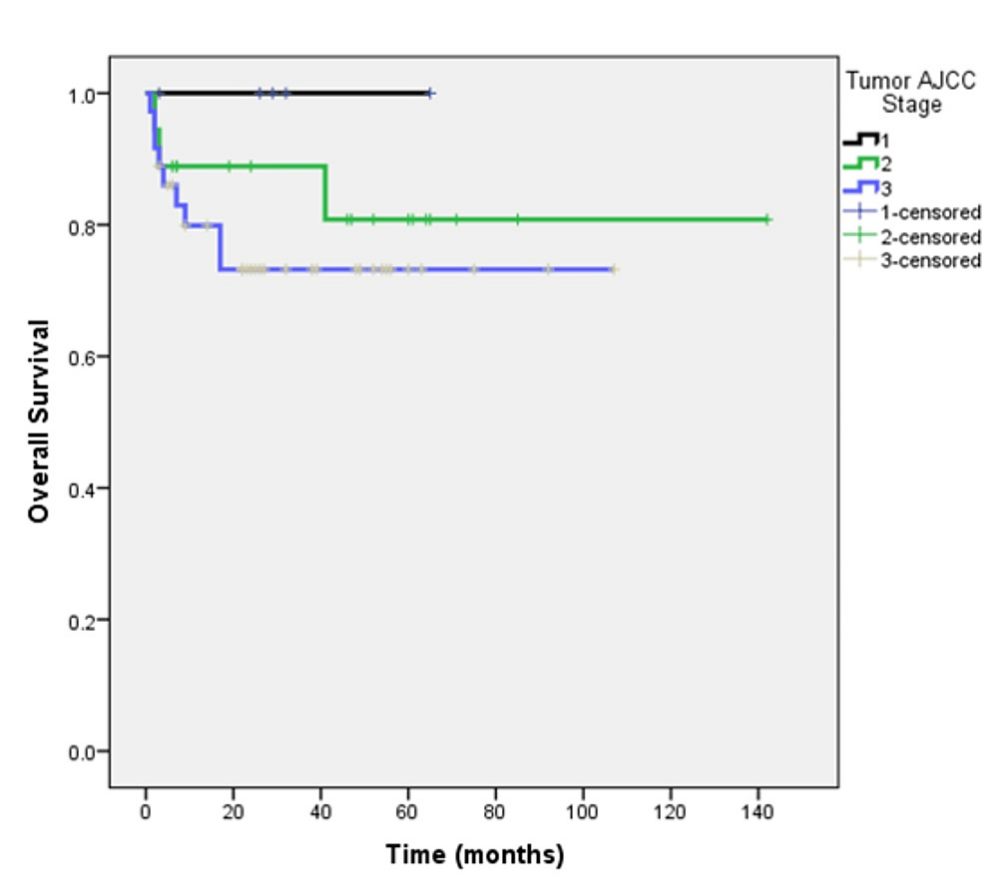
Overall survival of patients with non-metastatic according to tumor stage at the time of primary diagnosis (n=59 patients).

**Figure 3:**
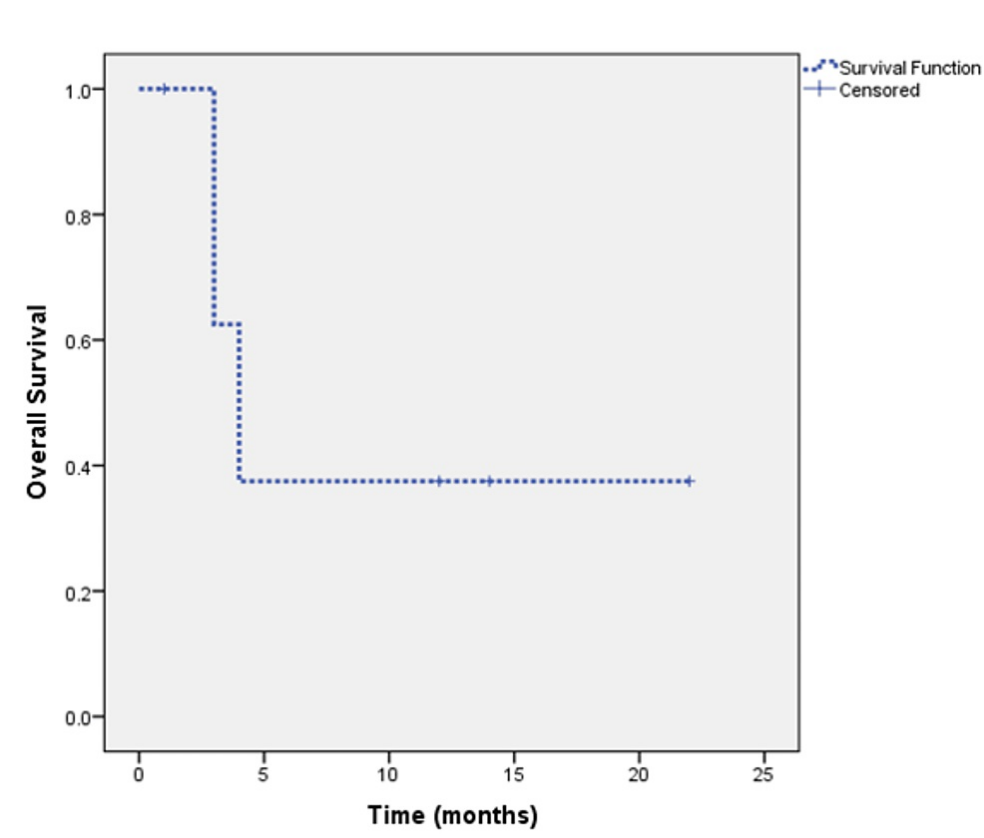
Overall survival of patients with stage IV metastatic disease at the time of primary diagnosis (n=59 patients).

The median follow-up of the total cohort was 32 months. At the time of analysis, 30 (44.1%) patients were alive and remained on regular follow-up, 17 (25%) patients died, and 21 (30.9%) patients lost to follow-up. The median overall survival (OS) was not reached and 71.6% remained alive at four years (Figure 1). Rates of survival were 100%, 80.8%, and 73.2% for patients with stages I, II, and III respectively (Figure 2). The median OS was four months for the nine patients who presented with stage IV metastatic disease of whom five had died and four patients remained alive at the time of analysis (Figure 3).

**Figure 4:**
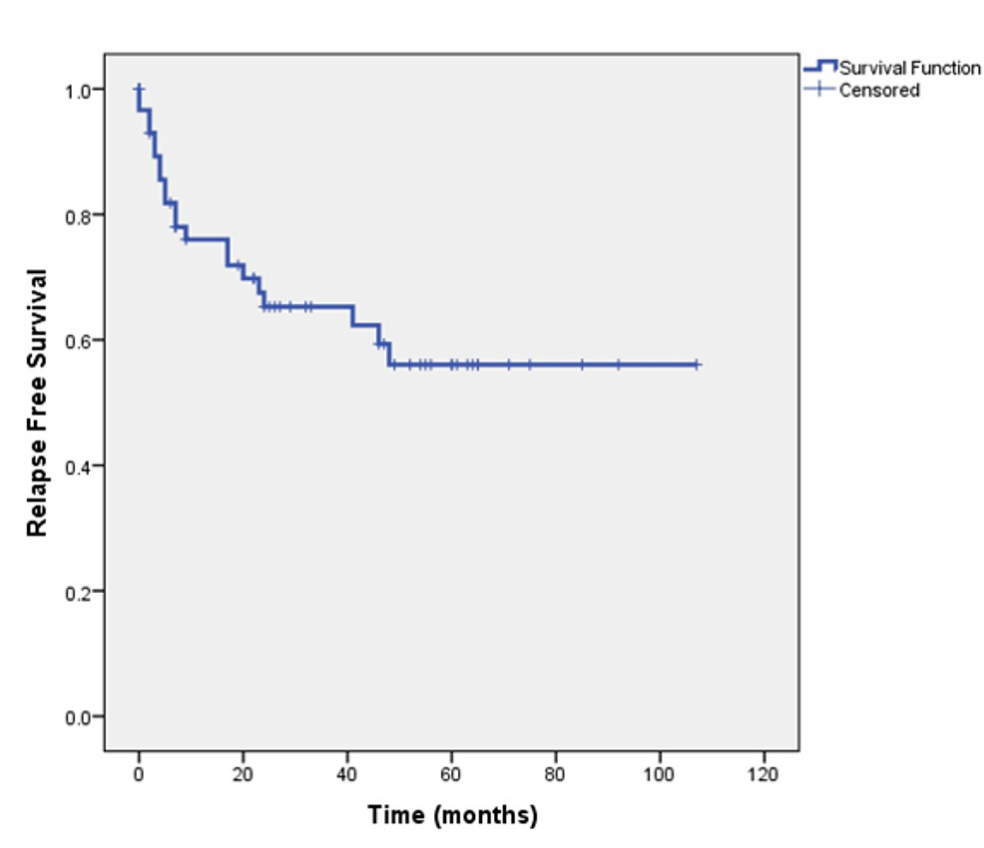
Relapse-free survival of patients with non-metastatic disease at the time of primary diagnosis.

**Figure 5:**
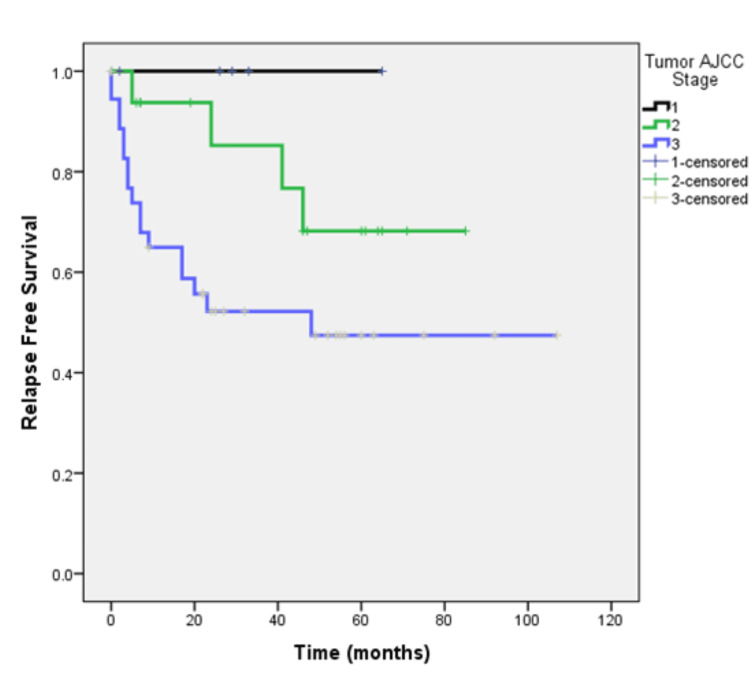
Relapse-free survival of patients with non-metastatic cancer according to tumor stage at time of primary diagnosis.

The tumor stage at diagnosis was I/II/III (non-metastatic) in 59 patients. After a median follow-up of 49 months, 21 (35.6%) of these patients experienced relapse. Median relapse‐free survival (RFS) was not reached and 56% were disease-free at four years (Figure 4). Rates of RFS were 100%, 68.2%, and 47.4% for patients with stages I, II, and III respectively (Figure 5).

## Discussion

Clinical characteristics

Of all the colorectal cancer patients 10-20% belong to the mucinous subtype [7]. Studies suggest that the ratio of female patients with mucinous subtypes is higher than the non-mucinous type [8-9]. However, our study cohort was composed of 54.4% males and 45.6% females, with a median age at diagnosis of 59 years, and a broad age range from 21 to 89 years. The slight male predominance is consistent with general colorectal cancer demographics in our region. The diversity of age groups affected by mucinous adenocarcinoma underscores the importance of vigilance across all demographics. The mucinous subtypes are found more commonly in the proximal colon [10]. In our study, 38.2% of the cases were right-sided and 51.5% were from the left side but these included the rectum as well and this reflects the higher incidence of diagnosis of rectal cancer in the region. Most tumors were classified as grade II (82.4%), consistent with the intermediate differentiation status often associated with the mucinous subtype [11]. The most common symptom at presentation was abdominal pain (55.9%) followed by per rectal bleeding and abdominal swelling and these are in line with presenting symptoms of all colorectal cancers [12]. Our study had several limitations. The collection sheet is self-administered, which may have introduced participant bias. In order to avoid this bias, we reviewed many data collection sheets regarding the same topic to assess the patient's condition. Another limitation was the refusal of some patients to participate in this study.

Treatment modality

In our study the management was in line with the standard established practice and surgical resection as expected was the primary potentially curative approach. However, a subset of patients was deemed unresectable due to locally advanced disease, or metastases identified at presentation. Notably of patients presenting with locally advanced rectal cancer, six patients underwent concomitant chemoradiotherapy followed by surgery, and four patients had upfront surgery The above reflects the important role of the wider multidisciplinary team to refine the complex treatment modalities that currently exist in order to achieve the best possible outcomes [13].

Survival outcomes

Studies suggest that patients with mucinous colorectal adenocarcinoma have a lower progression-free survival (PFS) rate (three-year PFS rate, 79.2% vs. 56.9%, respectively) and a shorter median OS (60.2 months vs. 48.4 months respectively [11-13]. Our study highlights a dynamic survival outcome based on stage of presentation The duration of median follow-up was 32 months. At the time of analysis, 44.1% of patients were alive and remained on regular follow-up, 25% had succumbed to the disease, and 30.9% were lost to follow-up. The median OS was not reached, and notably, 71.6% of patients remained alive at the four-year mark. This suggests that a substantial proportion of patients with the mucinous subtype achieve long-term survival despite the challenges that we face in diagnosing and treating these patients and highlights the importance of continued follow-up and surveillance. Prognosis based on staging is significantly different based on whether patients present with early (stages 1, 2, and 3) or advanced stage (stage 4). Park et al. showed that the five-year OS was significantly lower (81.4%) for stage I, II, and III mucinous colorectal adenocarcinoma patients as compared to non-mucinous colorectal adenocarcinoma patients (87.4%, P = 0.005) [14]. Maisano et al. treated stage IV colonic cancer patients with systemic chemotherapy which is the standard of care and observed a median OS of 8.0 and 18.0 months in patients with and without mucinous colorectal adenocarcinoma histology, respectively (HR, 1.99; 95% CI 1.26- 1.70; P = 0.03) [15]. When we stratified our results by the American Joint Committee on Cancer (AJCC) tumor stage it revealed variation in prognosis based on the staging. Patients with stages I, II, and III mucinous adenocarcinoma demonstrated survival rates of 100%, 80.8%, and 73.2%, respectively. In contrast, patients presenting with stage IV disease had a median OS of only four months, highlighting the grim prognosis associated with advanced-stage disease. For patients with early disease (stages I/II/III), the median RFS was not reached, and 56% remained disease-free at four years. However, the risk of relapse increased with more advanced stages, with RFS rates of 100%, 68.2%, and 47.4% for stages I, II, and III respectively. RFS rates declined with advancing stages, emphasizing the critical importance of early-stage detection and intervention in this subtype.

## Conclusions

This is a retrospective analysis of patients presenting with mucinous adenocarcinoma to a large tertiary teaching hospital in the Kingdom of Saudi Arabia which provides and substantiates valuable insights on clinic pathological parameters and survival outcomes of patients with this distinct subtype. It is well established that the prognosis of patients presenting with mucinous adenocarcinoma is poorer than for non-mucinous adenocarcinomas, our findings suggest that a significant proportion of patients can achieve extended survival, particularly when diagnosed at an early stage. It also confirms that patients with advanced disease continue to present formidable challenges with poor survival outcomes. Whilst our study contributes to the current understanding of mucinous adenocarcinomas of the colon, further research in molecular profiling and genomic testing and larger clinical trials with tailored treatments is necessary to refine treatment strategies and improve outcomes.
